# Atypical social behaviors in mouse models for Rett syndrome

**DOI:** 10.3389/fneur.2026.1744450

**Published:** 2026-04-15

**Authors:** Cesar Acevedo-Triana, Destynie Medeiros, Julia Lopes Gonçalez, Wei Li, Lucas Pozzo-Miller

**Affiliations:** 1 School of Psychology, Universidad Pedagógica y Tecnológica de Colombia, Tunja, Colombia; 2 Department of Neurobiology, University of Alabama at Birmingham, Birmingham, AL, United States; 3 Nash Family Department of Neuroscience, Icahn School of Medicine at Mount Sinai, New York, NY, United States; 4 Department of Pediatrics and Human Development, College of Human Medicine, Michigan State University, Grand Rapids, MI, United States

**Keywords:** BDNF, MeCP2, mouse models, Rett, social behavior

## Abstract

Social behavior depends on neural circuits that encode social identity, memory, and motivational value. These processes engage coordinated activity across hippocampal subregions, medial prefrontal cortex, thalamic and hypothalamic nuclei, as well as the mesocorticolimbic dopaminergic systems that regulate internal state, hierarchy, and social reward. In Rett syndrome and MeCP2-deficient rodent models, basic sociability is often preserved, alongside impairments in social memory, dominance behavior, aggression control, and flexible social responding, frequently accompanied by anxiety-like and sensorimotor disturbances. These behavioral phenotypes are associated with MeCP2-dependent molecular dysfunctions, including altered activity-dependent transcription, reduced BDNF–TrkB signaling, disrupted synaptic maturation, and altered network activity within prefrontal and hippocampal circuits. Together, these findings indicate that Rett-related social deficits reflect impaired integration and valuation of social information rather than a primary loss of social interest. Understanding how MeCP2 regulates the development and function of distributed social circuits may inform strategies to restore adaptive social behavior in Rett syndrome and related neurodevelopmental disorders.

## 1 Introduction

Rett syndrome (RTT) predominantly affects girls and women, with an incidence of approximately 1 in 10,000 live births, making it the second most common genetic cause of intellectual disability in females after Down syndrome (1). Typically, girls show typical early development followed by regression between 6–18 months, including loss of language and cognitive skills, alongside motor, respiratory, anxiety, seizure, and intellectual disability symptoms (2–11). In males, pathogenic *MECP2* variants on the only X chromosome result in a severe neonatal encephalopathy, typically lethal within the first 2 years of life (12, 13). Clinical severity in RTT varies with specific *MECP2* variants (14, 15). Eight common variants—R106W, R133C, T158M, R168X, R255X, R270X, R294X, R306C—account for over 60% of classic RTT cases. Variants such as R133C, R294X, and C-terminal truncations are generally milder, whereas large deletions and R168X are more severe, based on composite clinical severity scores encompassing regression onset, growth, motor and communication abilities, respiratory/autonomic dysfunction, and epilepsy (15, 16). Severity tends to increase with age within variant groups, reflecting the progressive nature of RTT (16). MeCP2 deficits across developmental periods disrupt maturation and regulation of excitatory and inhibitory circuits (12), and RTT is distinguished from idiopathic autism by a higher prevalence of epilepsy, linking altered network structure and function to clinical outcomes (17).

Loss of MeCP2 disrupts transcriptional programs that coordinate neuronal maturation, the stability of neuronal networks, and synaptic plasticity, in part through regulation of *Bdnf* and other activity-dependent genes. MeCP2 is a nuclear methyl DNA–binding protein that recognizes methylation-enriched genomic regions and recruits transcriptional repressors or activators depending on cellular context (5, 13). Through this epigenetic control, MeCP2 fine-tunes the expression of thousands of genes that govern neuronal differentiation, connectivity, and homeostatic plasticity (8, 12).

Loss of MeCP2 function destabilizes these regulatory networks across development. Neuropathological studies reveal reduced overall brain volume, smaller neuronal cell bodies, and fewer dendritic processes, without overt neurodegeneration or gliosis (12, 17). Cell density is increased in cortex, hypothalamus, and hippocampus, consistent with impaired dendritic maturation rather than neuronal loss (18). Neurochemical analyses show reduced dopamine, serotonin, and noradrenaline, which correlate with motor incoordination, stereotyped hand movements, and anxiety (17, 19), along with shortened and simplified dendritic spines in pyramidal neurons of frontal and motor cortices and hippocampus (12, 20).

Mouse models reproduce these molecular and structural phenotypes. Male *Mecp2* knockout mice develop tremor, irregular breathing, hypoactivity, and early death between 6 and 12 weeks, while female *Mecp2* heterozygous mice, due to the cellular ‘mosaicism’ resulting from X-chromosome inactivation, show delayed onset but progressive symptoms beginning at 4 to 6 months (21–23). Conditional reactivation of *Mecp2* in adult mice improves neurological function, indicating that symptoms arise from reversible circuit dysfunction rather than irreversible neuronal loss (24). Mutation type and genetic background further shape disease severity and trajectory (15, 25).

Social behavior is a fundamental feature of animal life, including humans, and is essential for living in families, groups, and communities. Individuals who share a social space must continuously adjust their behavior to current context, social rules, and internal need for contact, a process often described as social homeostasis (26, 27). For example, group living can enhance complex forms of cognition in mice, including discrimination of a conspecific in pain, social transfer of analgesia, and allogrooming as a prosocial response to distress (28, 29). At the same time, alterations in social environment exert powerful effects on brain function and behavior. Brief periods of isolation can increase subsequent prosocial behavior, whereas chronic isolation promotes territorial aggression and alters brain-derived neurotrophic factor (BDNF) protein levels (26, 30).

Because social animals naturally seek interaction, behavior in group-living species is shaped by social structure, including hierarchy and dominance relationships, which improves conditions inside the group and thus the survival chances of individuals (31, 32). Within these structures, individuals must recognize and remember others, evaluate whether to affiliate or compete, and flexibly shift between prosocial and aggressive strategies. Social recognition memory links prior interactions to current decisions, while aggression and dominance behaviors regulate rank and access to territory, mates, and food (33–37).

A useful conceptual framework divides social behavior into measurable components, including sociability, social recognition, aggression, and dominance. These components are supported by distributed neural circuits, with the hippocampus and medial prefrontal cortex playing central roles in encoding, evaluating, and guiding adaptive social behavior. The hippocampus contributes to social recognition and contextual representation, whereas the medial prefrontal cortex integrates this information with internal state and motivational signals to shape social decisions (33, 38–43).

The development and function of these circuits depend on precisely regulated synaptic growth, pruning, and plasticity shaped by early social experience and molecular regulators such as BDNF and activity-dependent transcription programs. Disruption of these processes can therefore have lasting consequences for social cognition, behavioral flexibility, and social motivation. Rett syndrome (RTT) provides a framework for understanding how perturbations of these systems lead to atypical social behavior. RTT is a neurodevelopmental disorder caused by pathogenic variants in *MECP2*, the gene encoding the methylated DNA-binding transcriptional regulator MeCP2, which regulates activity-dependent transcription of several genes controlling synaptic maturation and circuit function, including *Bdnf* (44–46). In individuals with RTT and in *Mecp2*-based mouse models, basic sociability is often preserved, whereas social memory, dominance behavior, aggression control, and flexible social responding are impaired (42, 47–52). This dissociation suggests selective disruption of social cognition and valuation rather than a generalized reduction in social approach.

Here, we consider how social behavior can be parsed into distinct components and how multiple brain circuits interact to support adaptive social behavior. We then examine how these circuits are assembled during development and how molecular mechanisms, including BDNF signaling and activity-dependent transcription, contribute to social cognition. Finally, we discuss how insights from RTT and MeCP2 function identify circuit and molecular targets for restoring social cognition and motivated social behavior.

## 2 Neuronal circuitry underlying social behaviors

**Figure 1:**
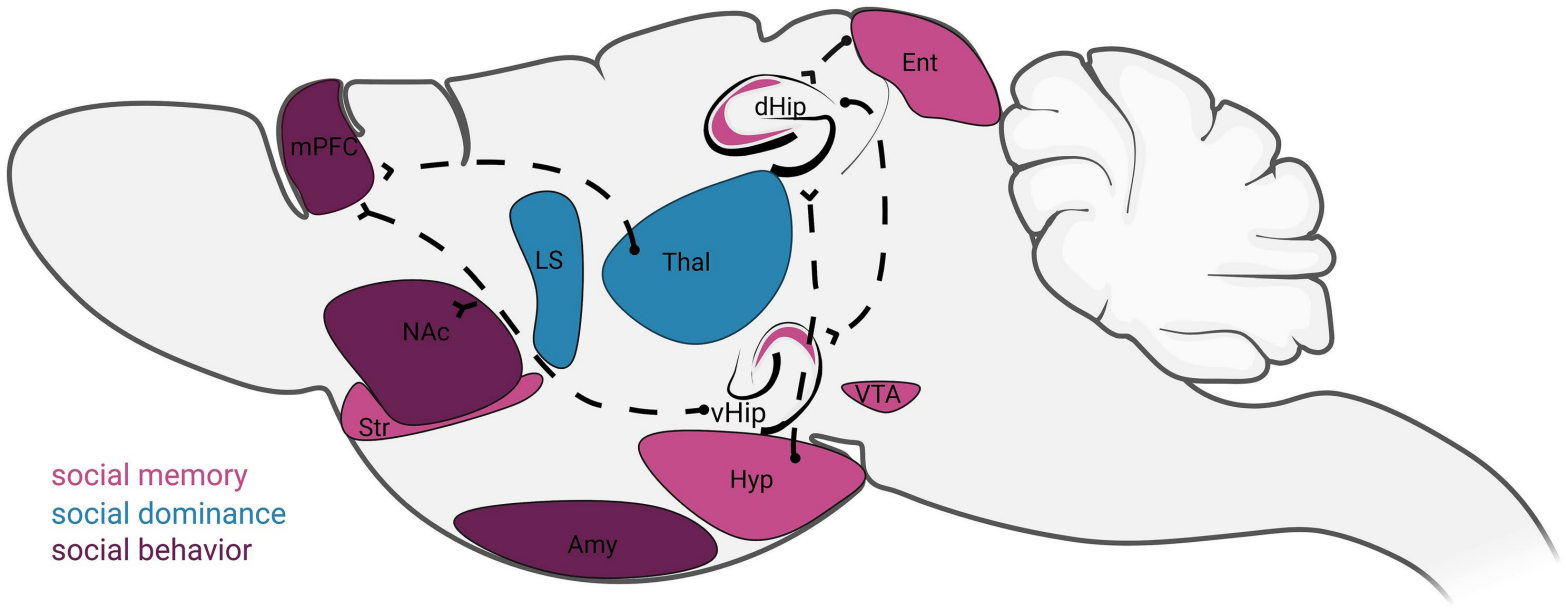
Rodent brain regions and neural circuits involved in the regulation of social behavior, social memory, and social dominance. Schematic sagittal view of the rodent brain highlighting key regions and pathways implicated in social behavior (purple), social memory (pink), and social dominance (blue). Shown regions include the medial prefrontal cortex (mPFC), nucleus accumbens (NAc), striatum (Str), lateral septum (LS), thalamus (Thal), dorsal hippocampus (dHip), ventral hippocampus (vHip), entorhinal cortex (Ent), hypothalamus (Hyp), ventral tegmental area (VTA), and amygdala (Amy). Dashed lines indicate major functional and anatomical connections between these regions that support social information processing, memory encoding and retrieval, and the regulation of dominance-related behaviors. Colors denote the primary social functions most strongly associated with each region, though many structures contribute to multiple aspects of social behavior.

Social behavior relies on distributed neural circuits that transform sensory cues, internal state, and prior experience into adaptive actions. In rodents, social encounters recruit coordinated activity across cortical and mesolimbic dopaminergic regions, each contributing distinct but interacting functions (Figure 1). Together, these circuits support social recognition, valuation, motivation, dominance, and aggression, enabling flexible behavioral responses across contexts (34, 40, 41).

Social experiences activate distributed neuronal ensembles across the brain. Neuronal activity mapping using the immediate early gene *c-fos* reveals robust activation of medial prefrontal cortex (mPFC), hippocampus, and amygdala following social interactions, consistent with engagement of large-scale networks during social recognition. Whole-brain c-Fos immunostaining further demonstrates that distinct social contexts recruit partially overlapping but dissociable neural patterns. For example, male–female interactions engage olfactory tubercle, nucleus accumbens (NAc), striatum, prefrontal and insular cortices, mediodorsal thalamus, ventral subiculum, and dorsal raphe, whereas male–male encounters engage fewer of these regions (53).

Technological advances have enabled direct measurement of neuronal activity during social behavior. Genetically encoded sensors of intracellular calcium concentration provide indirect readouts of neuronal activity, whose fluorescent signals can be monitored in freely behaving animals using ‘bulk’ population recordings from single sites by fiber photometry or cellular level imaging using miniature head-mounted fluorescence microscopes (54–58). Recordings from dopamine neurons in the ventral tegmental area (VTA) show increased activity during social interaction compared with object exploration, supporting a role for reward circuitry in social behavior (40). Imaging studies in hippocampus and mPFC further identify neuronal ensembles encoding social novelty and familiarity, linking population dynamics to social memory and decision making (59–61).

### 2.1 mPFC as an integrative hub

The rodent mPFC serves as an integrative hub that coordinates information relevant to social cognition, motivation, and behavioral flexibility. Subregions of the mPFC, including the anterior cingulate cortex, prelimbic cortex, and infralimbic cortex, contribute to higher-order social functions such as representation of conspecific pain, social transfer of affective states, empathy-like behaviors, and evaluation of social rank (28, 36, 62–65).

These functions depend on extensive long-range connectivity. The mPFC receives convergent inputs from ventral hippocampus, amygdala, mediodorsal thalamus, hypothalamus, and basal ganglia, enabling integration of contextual, emotional, motivational, and internal-state information. Ventral hippocampal projections support social recognition and memory (42, 66), while amygdalar inputs convey information related to sex recognition, salience, and threat (67). Mediodorsal thalamic and hypothalamic afferents encode social rank and physiological state, and basal ganglia inputs influence motivation and action selection during social interaction (35–37, 41).

Both excitatory pyramidal neurons and inhibitory interneurons within the mPFC encode social information. Prelimbic pyramidal neurons form ensembles that are selectively activated or suppressed by social novelty and salience, and single-unit recordings reveal neurons whose firing rates predict social engagement (60, 68). *In vivo* calcium imaging studies further demonstrate that distinct excitatory neuronal populations respond preferentially to social versus nonsocial stimuli. In *Mecp2* heterozygous female mice, these representations are degraded, with reduced activity and impaired differentiation of social ensembles despite preserved sensory processing (52, 59). In male *Mecp2* knockout mice, mPFC social representations are similarly disrupted, characterized by reduced population activity (50). Similar impairments in social encoding have been observed in *Cntnap2* knockout mice, indicating that disrupted mPFC population dynamics can underlie social deficits across preclinical models for neurodevelopmental disorder (68).

Inhibitory interneurons, particularly parvalbumin-expressing interneurons, play a critical role in stabilizing mPFC network activity during social behavior. Experimental manipulation of pyramidal neuron excitability or inhibitory tone in the mPFC produces reversible deficits in sociability, while selective activation of parvalbumin interneurons rescues social impairments caused by ventral hippocampal dysfunction or adolescent social isolation (65, 69–72). Consistent with this role, lesions of the mPFC selectively impair long-term social recognition without disrupting basic social preference (73). Similarly, in male *Mecp2* knockout mice, aberrantly elevated parvalbumin interneuron activity during interactions with familiar conspecifics may represent a key driver of impaired social memory (50).

### 2.2 Hippocampal circuits for social and contextual recognition and memory

The hippocampus supports multiple memory domains, including social memory (74). Specialized subfields contribute to social cognition: dorsal CA2 pyramidal neurons are critical for encoding and recalling conspecific identity without affecting locomotion, anxiety, or standard spatial and object recognition tasks (75, 76), while ventral CA1 stores engrams of familiar mice and ventral CA3 is required for encoding but not recall (61, 76, 77). CA2 sharp-wave ripples during non-REM sleep consolidate social memories, such that enhancing ripples strengthens memory for a previously unfamiliar conspecific, whereas disrupting them impairs recall (78).

Inputs from lateral entorhinal cortex convey social-stimulus identity, and vasopressin-containing projections from the paraventricular nucleus of the hypothalamus enhance CA2-mediated social memory (79, 80). Hippocampal outputs to mPFC and NAc shell modulate social recognition (61), while chemogenetically inhibiting hyperactive ventral hippocampal inputs to mPFC rescues social memory in male *Mecp2* knockout mice (42).

### 2.3 Circuits for social aggression and dominance

Circuits supporting social recognition overlap with those mediating social aggression and hierarchy. The medial amygdala, bed nucleus of the stria terminalis, ventromedial hypothalamus ventrolateral subdivision, and premammillary nucleus receive olfactory input and can evoke or suppress aggression, with modulation by the lateral septum and mPFC (34). Projections from the mPFC to hypothalamic aggression-related nuclei coordinate agonistic behaviors and competitive interactions (33, 81).

The mPFC is essential for social hierarchy, which depends on social recognition and evaluation of competitive outcomes. Neuronal activity in the prelimbic mPFC correlates with dominance expression, decision making, and success in tube-test competitions (35–38, 82). Mediodorsal thalamic projections to mPFC are sufficient to bias competition outcomes, and chronic manipulation of this pathway alters hierarchy formation (37, 82). The forebrain–thalamocortical pathway, where the mediodorsal thalamus (MDT) acts as a hub receiving inputs from orbitofrontal cortex and basal forebrain, and projecting to the caudal anterior cingulate cortex (cACC), was shown to regulate competitive performance during hierarchy formation. This circuit is linked to changes in *Trpm3* expression and synaptic properties, with MDT projections driving inhibition of cACC pyramidal cells in higher ranked individual mice (83). Mitochondrial function within the NAc also contributes to hierarchical stability (82). Classic lesion and activation studies further support roles for anterior cingulate and prelimbic cortices in social engagement and memory (84–86). Beyond neurons, astrocytes regulate mPFC network states and hierarchy: astrocytic calcium dynamics correlate with dominance behaviors, and their chemogenetic activation increases rank in subordinates while silencing lowers rank in dominants (87).

### 2.4 Hormonal and developmental modulation

Hormonal and developmental factors further shape these networks. Testosterone, estradiol, and glucocorticoids modulate activity in hippocampus, mPFC, amygdala, and hypothalamus, influencing aggression, dominance, and responses to social stress. Testosterone levels typically rise after competition and correlate with dominance, whereas castration reduces, and hormone replacement restores, dominance behaviors (82, 88–92). Under baseline conditions with sufficient resources, glucocorticoid levels are comparable across ranks, but when access is limited, subordinates show elevated glucocorticoids reflecting chronic stress (86).

Developmental adversities and early life stress reshape these circuits. Elevated corticosterone during critical periods alters maturation of hippocampus, mPFC, and amygdala, producing long-lasting effects on social memory and hierarchy formation (35, 37, 42, 67, 91). For example, limited bedding and nesting followed by social defeat leads to lower adult dominance scores and transcriptional changes in GABAergic and glutamatergic neurons, accompanied by altered ventral hippocampal responses to social stress (93).

Together, these findings indicate that social behavior arises from coordinated activity across hippocampal and mPFC networks, extended amygdala and hypothalamic nuclei mediating aggression and defense, and mesolimbic dopamine and striatal circuits that assign value and guide motivation.

## 3 Developmental maturation and modulation of social circuits

Early social experience establishes the foundations of social recognition, motivation, and regulation of behavior. The first manifestations of social memory arise through caregiver and sibling interactions, mediated primarily by olfactory cues. Hippocampal CA2 neurons support this recognition as early as postnatal day 3 (94). Pups preferentially investigate their caregiving mother, but after weaning this preference shifts toward novelty, suggesting that social novelty preference emerges developmentally while CA2 continues to mediate recognition of familiar individuals (94). Inhibition of CA2 in adulthood prevents maternal recognition, indicating a persistent role for CA2 in social memory across life (75, 76, 78, 79, 94).

### 3.1 Dopaminergic modulation of social behavior

The dopaminergic system contributes to social preference and social memory and is disrupted in RTT (19, 95, 96). Projections from ventral CA1 to NAc modulate social discrimination (61), and mPFC projections to NAc contribute to social memory recognition (97). The activity of dopaminergic neurons in NAc rises during social approach, investigation, and consummatory behavior (98). Dopaminergic neurons in VTA show heightened activity during interactions with unfamiliar conspecifics, reinforcing social learning (99). Within mPFC, neurons expressing dopamine D2 receptors are activated during social exploration (100). Chemogenetic activation of ventral striatal dopaminergic neurons acutely increases prefrontal dopaminergic activity during social encounters, whereas prolonged activation diminishes dopaminergic responses to social stimuli (101). Together, these findings show that dopamine regulates social motivation and learning through interconnected ventral hippocampal, prefrontal, and striatal pathways.

### 3.2 Epigenetic translation of social experience

Early social exposures, including maternal care, separation, abuse, neglect, and environmental enrichment, influence brain function through epigenetic mechanisms such as DNA methylation and histone modification. MeCP2, a methylated DNA reader, translates these experiences into durable transcriptional changes in genes including *Bdnf*, *Crh* (corticotropin-releasing hormone), and *Avp* (arginine vasopressin), thereby altering stress responsiveness and social behavior (102). The intracellular signaling cascade engaged by BDNF binding to its tropomyosin kinase B (TrkB) receptor acts as a major downstream mediator linking reward, stress, and synaptic maturation, with its expression and epigenetic regulation remaining environmentally sensitive into adolescence and adulthood (102).

### 3.3 Effects of early life stress and maternal separation

Prolonged maternal separation heightens anxiety- and depression-like behaviors, whereas brief separations can blunt stress responses, particularly in males, by enhancing maternal and pup interactions (102). Extended separation alters dopaminergic signaling, reducing 3,4-dihydroxyphenylacetic acid (DOPAC), a primary dopamine metabolite that reflects dopamine turnover (103). It also increases dopamine D1 receptor binding in NAc core and caudate-putamen, elevates dopamine D3 receptor mRNA in NAc shell, and decreases dopamine transporter levels in striatal regions, but not in VTA or mPFC (103).

### 3.4 Epigenetic regulation of BDNF and stress-related genes

BDNF signaling links social experience to neurodevelopment. Adult social defeat correlates with increased di-methylation of the lysine residue at position 27 in histone 3 (H3 K27) at the *Bdnf* promoters III and IV, as well as altered histone de-acetylase expression (104). During development, BDNF supports maturation of dopaminergic, serotonergic, and GABAergic neurons (105–108), and its expression remains responsive to environmental modulation throughout life. Postnatal abuse decreases prefrontal *Bdnf* expression through promoter IV hyper-methylation (109), while adult social defeat reduces hippocampal *Bdnf* promoter III and IV transcripts, increases 3 K27 di-methylation, and alters the expression levels of two histone de-acetylases, HDAC2 and HDAC5, paralleling changes observed in human depression (102).

Mechanistically, maternal separation induces CaMKII-dependent MeCP2 phosphorylation by postnatal day 10 at a CpG island in the *Avp* gene (CGI3), reducing methylation and increasing *Avp* mRNA, whereas brief handling enhances NRSF binding to the *Crh* gene by postnatal day 9, lowering *Crh* mRNA (102). These findings demonstrate how early-life social environments sculpt gene expression and neural circuits through MeCP2-dependent epigenetic processes.

## 4 BDNF as a molecular gatekeeper of social plasticity: the role of BDNF in synaptic growth, circuit maturation, and social behavior

### 4.1 Neurotrophins and neural development

**Figure 2:**
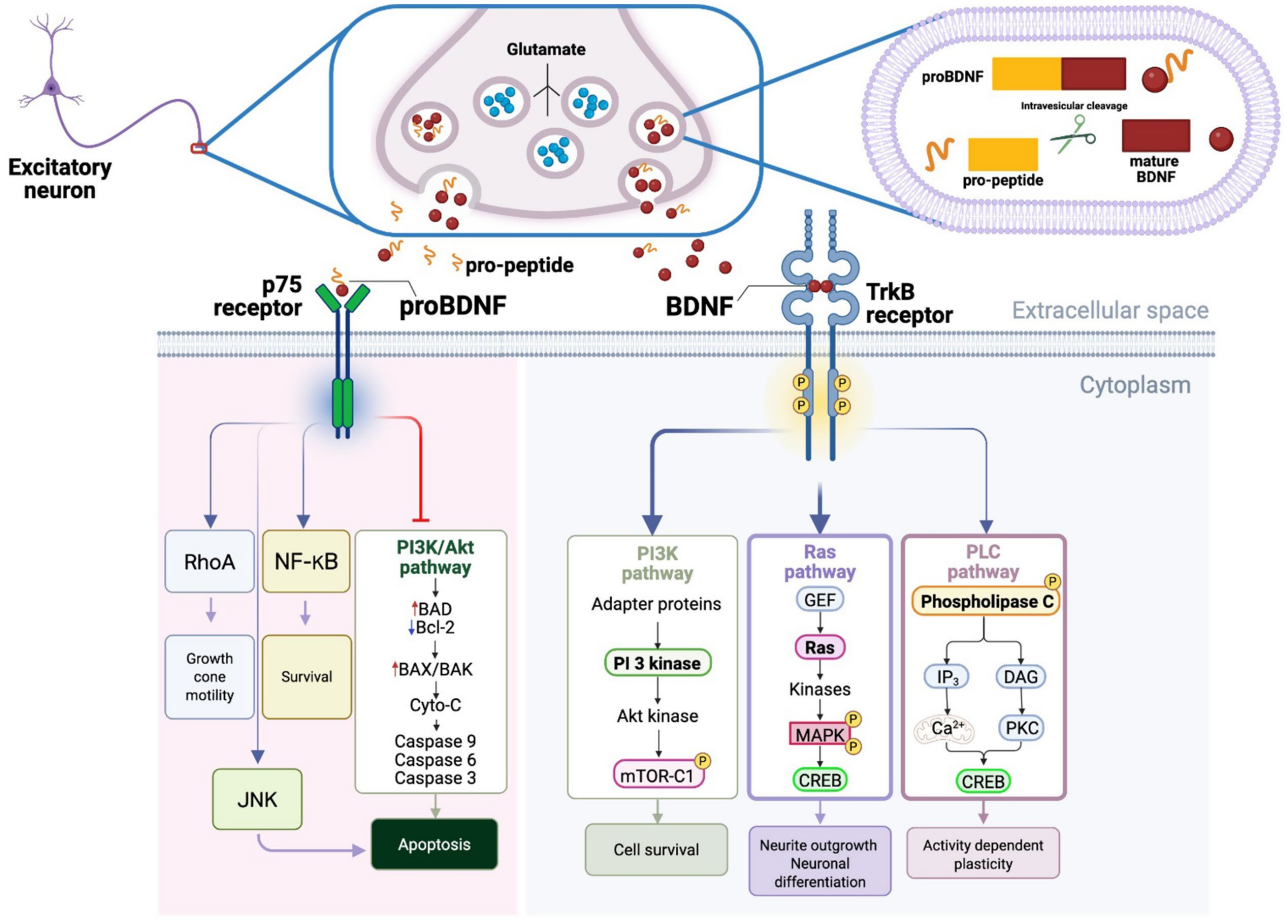
BDNF–TrkB signaling pathways. BDNF is synthesized in excitatory neurons as a precursor (proBDNF) and packaged into large dense-core vesicles for activity-dependent release near synapses. Proteolytic processing generates mature BDNF and the BDNF pro-peptide, which may be co-released into the synaptic/extracellular space alongside glutamate from small clear synaptic vesicles. In the extracellular space, proBDNF (and, in some contexts, the pro-peptide) can preferentially engage the p75^NTR^ receptor, activating pathways linked to structural remodeling and stress-related responses (e.g., RhoA, JNK, NF-κB) and, under specific conditions, pro-apoptotic programs. By contrast, mature BDNF binding to the TrkB receptor promotes their dimerization and tyrosine autophosphorylation (yellow “P” circles), creating docking sites for adaptor/effector proteins. These activated TrkB receptors engage three major signaling cascades: (1) The PI3K pathway, in which adapter proteins recruit/activate PI3K, leading to Akt activation and downstream mTORC1 signaling to support cell survival; (2) the Ras/MAPK pathway, where a GEF activates Ras, triggering a kinase cascade culminating in MAPK activation and phosphorylation of CREB, promoting neurite outgrowth and neuronal differentiation; and (3) the PLC pathway, where phospholipase C (PLC) generates IP_3_ and DAG, elevating intracellular Ca^2+^ and activating PKC, which converge on CREB phosphorylation to drive activity-dependent plasticity. Bold arrows indicate TrkB branches with the most consistent pathway-resolved links to social-behavior phenotypes (ERK/MAPK and PI3K/Akt/mTOR) across social stress/susceptibility models, whereas the thin arrow highlights PLCγ signaling, which is implicated in plasticity but is less often isolated as the primary causal driver *in vivo*. For an in-depth recent review on BDNF signaling, see Barde (221).

Mammalian neurotrophins such as nerve growth factor (NGF), BDNF, neurotrophin-3 (NT-3), and NT-4 orchestrate key stages of neural development, including neuronal proliferation, differentiation, axonal growth, and synaptogenesis (17, 110–112). Among these, BDNF is the most abundant in hippocampus and cortex, is produced by neurons and astrocytes, and signals through high-affinity TrkB receptors with intrinsic tyrosine kinase activity (Figure 2) (112, 113). BDNF is also unique among neurotrophins in that its release from dense-core vesicles depends on the calcium-dependent regulated pathway (113).

### 4.2 Molecular processing and TrkB signaling

BDNF is synthesized as proBDNF in the endoplasmic reticulum, sorted through the Golgi apparatus, packaged into large dense-core vesicles, and proteolytically cleaved to mature BDNF, which is released mostly in an activity-dependent manner; proBDNF can also be secreted and cleaved in the extracellular space to mature BDNF (114, 115). Mature BDNF binds to TrkB receptors at both pre- and postsynaptic sites and surrounding glial cells (110, 112).

Upon ligand binding, TrkB receptors dimerize and autophosphorylate, recruiting SH2-containing effectors and activating three principal signaling pathways: (1) MAPK/ERK, supporting transcriptional and translational regulation of synaptic proteins; (2) PI3K/mTOR, regulating cellular growth and metabolism; and (3) PLC, which generates IP3 to mobilize intracellular calcium stores via IP3 receptors and diacylglycerol to activate PKC as well as TRPC3-containing non-selective cationic channels with high calcium permeability (Figure 2) (110, 112). Together, these cascades modulate intracellular calcium, gene expression, neuronal excitability, neurotransmitter release, and synaptic strength, enabling the BDNF modulation of experience-dependent plasticity and memory formation (113, 114, 116, 117).

### 4.3 Epigenetic regulation of BDNF and social experience

Building on these developmental and stress-dependent mechanisms, BDNF expression remains epigenetically regulated across the lifespan in response to social experience. During early postnatal life, BDNF supports the maturation of glutamatergic, GABAergic, dopaminergic, and serotonergic neuronal populations (105–108), and its expression remains sensitive to environmental input throughout life. Adult social defeat correlates with increased H3 K27 di-methylation at *Bdnf* promoters III and IV and altered histone de-acetylase expression (104). Postnatal abuse reduces prefrontal *Bdnf* expression through promoter IV hypermethylation (109), and adult social defeat decreases hippocampal *Bdnf* promoter III/IV transcripts, accompanied by altered HDAC2/5 expression and increased H3 K27 di-methylation, paralleling epigenetic signatures observed in human depression (102).

### 4.4 BDNF–TrkB signaling as a gatekeeper of circuit plasticity

Through these mechanisms, BDNF–TrkB signaling acts as a molecular gatekeeper of circuit plasticity. By regulating gene transcription, protein synthesis, intracellular trafficking, synaptic growth, and long-term potentiation, BDNF enables coordinated maturation of hippocampal, cortical, and limbic networks essential for social cognition and emotional regulation (118–123). Disruption of this pathway impairs dendritic arborization, maturation of presynaptic terminals, and activity-dependent synapse stabilization, contributing to neurodevelopmental and psychiatric disorders (112).

### 4.5 BDNF signaling in psychiatric and social disorders

Altered BDNF–TrkB signaling is associated with depression (124, 125), anxiety (126), schizophrenia (127, 128), and posttraumatic stress disorder (128–130). In rodent models, social defeat reduces Bdnf mRNA and protein levels in orbitofrontal cortex, mPFC, and hippocampus, while in some cases elevating BDNF levels in amygdala and mesolimbic regions; co-housing can mitigate those hippocampal reductions (129, 131, 132).

Social stress broadly alters BDNF–TrkB signaling. Adolescent social isolation increases *Bdnf* mRNA and H3 acetylation at the *Bdnf* locus, elevating BDNF protein in mPFC while reducing hippocampal BDNF (30). Early-life stress modifies prefrontal *Mecp2* and *Bdnf* expression and increases cocaine cue motivation (133). Loss of *Bdnf* promoters I and II in the hypothalamus reduces sexual receptivity and impairs maternal care, with deletion of *Ntrk2* (encoding TrkB) in oxytocin neurons partially reproducing these deficits (134). In males, hippocampal BDNF supports social learning during hierarchy formation, with dominant individuals exhibiting higher expression (135). BDNF signaling in the ventral CA1–mPFC circuit also contributes to fear learning, with reduced BDNF impairing extinction recall, while contextual fear extinction elevates mPFC *Bdnf* mRNA, potentially priming excitatory synapses for plasticity; ventral CA1 neurons encode social identity, sex, and strain information correlated with theta-based temporal coding (113, 136–139).

## 5 Rett syndrome and its alterations in social behavior

Disruption of BDNF–TrkB signaling is a defining consequence of *Mecp2* loss, leading to dysregulation of transcriptional programs required for synaptic maturation and circuit refinement. These molecular and cellular alterations propagate across hippocampal, prefrontal, and striatal networks, contributing to the behavioral and cognitive impairments that characterize RTT.

### 5.1 Clinical and behavioral features of RTT

RTT presents with profound but variable motor, cognitive, and affective impairments. Core motor symptoms include loss of purposeful hand use, gait disturbances ranging from mild imbalance to complete loss of walking, and stereotyped hand movements such as wringing, clasping, clapping, mouthing, and washing-like automatisms (10, 140, 141). In rodent models, male *Mecp2* null rats and knockout mice show reduced forelimb grip strength and decreased locomotor activity in novel arenas, along with shorter stride length and atypical gait progression (50, 142, 143). Female *Mecp2* heterozygous mice show more shuffling locomotion, reduced velocity and shorter distance traveled during disease progression (49). Two-photon imaging during treadmill walking shows that male *Mecp2* knockout mice fail to scale stride length or total distance with speed and make fewer strides at each speed; in contrast, wildtype mice show motor cortex activity that correlates with speed and becomes more efficient with training, patterns absent in male *Mecp2* knockout mice (144). Motor learning deficits on the rotarod in female *Mecp2* heterozygous mice are improved by the partial TrkB agonist PTX-BD4-3 (145), linking motor dysfunction to impaired BDNF signaling (25, 146).

Anxiety is common in RTT and increases with age and with milder *MECP2* variants, whereas overall clinical severity shows the opposite relationship (147–149). In mice, anxiety-related phenotypes depend strongly on genotype, age, strain, and the affected cell population. Female *Mecp2* heterozygous mice show fluctuating anxiety-like behavior in the open field between 3 and 7 months (150). Male R255X knock-in mice spend more time in the open arms of the elevated plus maze, an effect not observed in females (146). Conditional deletion of *Mecp2* in *Sim*-expressing hypothalamic neurons or *Th*-expressing dopaminergic neurons reduces center exploration, whereas deletion in *Pet1*-expressing serotonergic neurons increases activity and alters fear responses (19, 151, 152). Male mice expressing a non-functional truncated MeCP2 protein (Mecp2 308) show hyperreactivity and reduced center time rather than classic anxiety-like behavior in the plus or zero mazes (153–155). Together, these findings highlight the importance of cell type, circuit context, and genetic background in emotional regulation (25, 156).

Cognitive impairment is another hallmark of RTT. Intellectual disability is well documented (157, 158), and sensory memory, particularly for auditory stimuli, is reduced (159). Because many individuals lose purposeful hand use and speech, eye gaze is commonly used as a proxy for cognition. Individuals with RTT show intentional gaze but reduced attention, fewer fixations, and longer fixation durations in visual paired comparison tasks; seizures and altered EEG patterns further influence performance (160–162). In mice, hippocampal-dependent tests such as contextual and cued fear conditioning, novel object recognition and location, and passive avoidance reveal consistent learning and memory deficits across different *Mecp2* genotypes (163, 164). Examples include reduced contextual freezing in R255X males, impaired contextual fear and passive avoidance in *Mecp2* heterozygous females at 3 to 5 months, and poorer one-day contextual recall in *Mecp2* heterozygous females (25, 146, 165). Object location memory deficits in *Mecp2* heterozygous females are rescued by partial TrkB agonism, and cell type–specific *Mecp2* deletions bidirectionally alter cue- and context-related freezing, further linking MeCP2- and BDNF-dependent plasticity to cognitive function (19, 166).

Together, human and mouse studies converge on a phenotype that includes severe motor disability, variable anxiety, and broad cognitive impairment. These features are strongly shaped by genotype, genetic background, age, and affected circuitry, and they provide robust readouts for testing circuit-targeted interventions.

### 5.2 Alterations in social behaviors

Social behavior offers a particularly sensitive window onto internal state, cognitive capacity, and circuit function. In RTT, social anxiety and withdrawal are common, with difficulty engaging peers and reduced frequency of interactions compared with age matched controls (47, 48, 147, 167). At the same time, social interest is often preserved. Because motor and speech abilities are constrained, individuals rely heavily on prelinguistic signals, especially eye contact and gaze, to communicate (168, 169). Parent reports indicate that girls with RTT convey affect through vocalizations, movements, and gaze (170), and eye tracking shows preserved preference for social stimuli with a stronger bias toward the eyes than the nose or mouth relative to controls (169). These clinical observations have motivated the development of behavioral interventions and assistive tools focused on gaze-based communication and social skills (171–175).

Preclinical models recapitulate this dissociation between basic sociability and social cognition. *Mecp2*-deficient mice and rats show impaired social preference in the three-chamber assay, spending equal time with an empty chamber or a familiar conspecific, and they show impaired social memory by interacting equally with familiar and novel conspecifics (51). In a modified three-chamber task, female *Mecp2* heterozygous mice divide time equally between a novel mouse and an object and later between a familiar and a novel mouse, indicating deficits in both preference and recognition (52). Male *Mecp2* knockout mice retain social preference but lose social memory, similar to findings in MeCP2-308 males, which express non-functional truncated MeCP2 protein (42, 50, 176). Chemogenetic inhibition of the ventral hippocampus–medial prefrontal cortex pathway in male *Mecp2* KO mice rescues this deficit, implicating disrupted hippocampal–prefrontal communication (42). Notably, female *Mecp2* heterozygous mice also exhibit atypically elevated social aggression phenotypes alongside a trend toward impaired social memory, highlighting additional sex-specific disruptions of social behavior beyond basic sociability (49). Mild overexpression of human *MECP2* also yields intact preference but impaired memory in a modified task, suggesting that both too little and too much MeCP2 can disturb the network balance needed for social cognition (177). Genetic background further modulates outcomes, as female *Mecp2* heterozygous mice on different hybrid strains show altered social preference at 3 and 5 months (25).

Social dominance and aggression behaviors are also affected. Deletion of *Mecp2* in *Sim1*-expressing hypothalamic neurons increases tail rattling and attacks during open field exposure, while conditional deletion in *Pet1*- or *Fev*-expressing serotonergic neurons elevates aggression in the resident intruder test (19, 151). In tube test competitions, wildtype mice tend to retreat from MeCP2-308 males, indicating altered dominance dynamics (154). MeCP2-308 males also show hyper-reactive escape and defensive behaviors in predator defense paradigms (153). Consistent with these findings, MeCP2 protein levels in mice and *MECP2* polymorphisms in schizophrenia patients are associated with measures of social aggression (178).

Together, human and animal data indicate prominent disturbances in social engagement, preference, memory, and aggression in RTT. These phenotypes reflect disruption of hippocampal–prefrontal, hypothalamic, and monoaminergic circuits, and they provide rigorous outcome measures for testing circuit based and molecular interventions aimed at restoring social function (42, 52, 177).

### 5.3 MeCP2 regulates *Bdnf* expression and activity dependent transcription

Across development, MeCP2 tightly regulates *Bdnf* expression in an activity-dependent manner. *Bdnf* expression is relatively low before birth and rises postnatally in parallel with MeCP2 protein levels, synaptogenesis, and developmental cell death (110, 117). In a dual-operation model, MeCP2 represses *Bdnf* at rest by binding methylated CpG sites at *Bdnf* promoters and recruiting corepressors. Neuronal activity induces MeCP2 phosphorylation and release from DNA, followed by SIRT1-dependent chromatin remodeling and transcriptional initiation at *Bdnf* promoters (110).

MeCP2 also cooperates with CREB1 at exon IV of Bdnf to enhance transcription, while BDNF in turn regulates MeCP2 through feedback loops involving miR-132, a negative regulator of MeCP2. These interactions form a homeostatic system in which MeCP2 and BDNF jointly stabilize activity-dependent plasticity (3, 110, 114). Consistent with this framework, *Bdnf* transcription depends on calcium entry through voltage-gated channels, CaMKIV activation, and CREB phosphorylation (179–181). In *Mecp2*-deficient mice, this regulatory cycle is disrupted: neuronal activity is reduced, for example in layer 5 pyramidal neurons of somatosensory cortex, and BDNF levels decline in whole brain homogenates, hippocampus, and brainstem (182–187). These changes are accompanied by reduced brain and neuronal size, altered spine structure, and synaptic phenotypes resembling partial *Bdnf* deficiency (12, 13, 44).

Experimental elevation of BDNF ameliorates multiple *Mecp2*-related phenotypes. Overexpression of *Bdnf* in mice restores neuronal morphology and synaptic output, effects that require TrkB signaling (183, 188). In cultured neurons, plasmid-driven BDNF expression improves dendritic complexity and synaptic transmission (189). Pharmacological strategies that enhance BDNF or TrkB activity, including the ampakine CX546 and fingolimod, improve respiratory output and other physiological deficits (190–193). TrkB agonists such as LM22A-4 reduce brainstem hyperexcitability, decrease apneas, stabilize hippocampal transmission allowing object location memory, restore spine phenotypes, and reduce aggressive behaviors in female *Mecp2* heterozygous mice (49, 145, 166, 186, 194).

Taken together, these findings position BDNF insufficiency and impaired TrkB signaling as central contributors to RTT pathology and provide a mechanistic bridge between MeCP2 loss, altered activity-dependent transcription, defective synaptic plasticity, and atypical social behavior.

## 6 Integrative perspectives and future directions

Social behavior can be separated into two related components. The first is recognizing and recalling others, or social memory. The second is being motivated to seek and maintain interaction, or social motivation (42, 75, 76, 195–198). These processes rely on overlapping circuits that include the ventral hippocampus–mPFC pathway, midline thalamic and hypothalamic inputs that signal internal state, and mesocorticolimbic dopamine systems that assign reward value to social contact (42, 61, 71, 76, 199). In typical development, these networks integrate social identity, affective meaning, rank, and expected outcomes to generate adaptive, goal directed social behavior (68, 200–202).

In RTT individuals and *Mecp2*-deficient mice, basic sociability often appears relatively preserved in simple tasks, with consistent impairments in social memory and context appropriate social responses are consistently impaired (42, 47, 48, 50, 203–205). This pattern suggests that MeCP2 loss selectively disrupts how social information is evaluated, stored, and transformed into future choices rather than producing a global reduction in social interest. Historically, rodent assays have focused on proximity-based measures of sociability or recognition, such as time spent near a conspecific in the three-chamber test (60, 75, 202, 206–208). However, these measures can reflect place preference, novelty seeking, or general exploration rather than true social engagement (209). Social conditioned place preference has been used to assess prosocial reinforcement, particularly during juvenile play, but results vary across studies (210, 211).

Operant social self-administration tasks address some of these limitations. In these paradigms, an animal performs an instrumental action, such as a lever press or nose poke, to obtain brief social access (197). The rate and persistence of responding indicate how much effort the subject is willing to invest to gain social contact (210, 212, 213). This framework treats social interaction as a contingent reward and enables trial-by-trial analysis of learning and reinforcement, increasing ecological validity and allowing dissection of social motivation (209, 210, 212). These tasks can be combined with circuit-specific recording and manipulation. Calcium imaging of the ventral hippocampus to mPFC projection during operant social tasks can test whether activity in this pathway tracks motivation to work for social access in addition to social recognition (42). Optogenetic inhibition of this pathway during the task is expected to reduce responding for social contact, whereas activation should enhance it, indicating a causal contribution to social pursuit rather than identity coding alone.

A complementary approach is to monitor dopamine dynamics during motivated social behavior (39, 40, 43, 212, 214). Genetically encoded fluorescent dopamine sensors allow real-time tracking of dopamine release in mPFC or striatal targets during trial initiation, action execution, and receipt of social reward. This approach can reveal how dopamine signals moment-to-moment value of social contact and how these signals coordinate activity between hippocampal and prefrontal nodes. Applying this combined behavioral and circuit strategy to *Mecp2*-deficient mice can clarify whether RTT-like social phenotypes arise from impaired assignment of reward value to social contact, failure to stably encode social partners, or both. Because MeCP2 loss alters activity-dependent transcription and BDNF–TrkB signaling (44–46, 182, 214, 215), this framework also enables direct testing of whether restoring circuit function or neuromodulatory tone can rescue motivated social behavior. The goal is to move beyond global descriptions of reduced social behavior and identify which step in the chain from perception to valuation to goal-directed pursuit is disrupted and amenable to intervention.

Based on recent reports and results from our lab, social behavior should be analyzed carefully based on sex. For example, female mice can form robust dominance hierarchies, yet the behavioral dynamics and circuit rules supporting social status can differ from that in male mice. Recent studies have shown that in female mice, hierarchies can be linear and stable, but rank consolidation tends to be slower and the “winner effect” weaker than in male mice, paralleling impaired synaptic plasticity in the thalamus-mPFC pathway, with stronger PV-interneuron gating of dominance-related learning (216, 217). More broadly, the sexual dimorphism in the aggression circuitry (e.g., sex-biased excitation–inhibition balance) underscores the fact that apparently similar social phenotypes could be implemented by different brain circuits in male and female mice (218). Therefore, future translational studies linking social rank to stress vulnerability or neuropsychiatric outcomes should avoid generalizations solely based on observations made in male mice, prioritizing sex-stratified experimental design with proper estimation of sample sized based on effect size in each sex, with mechanistic tests tailored to sex-specific circuit predictions.

## 7 Summary and conclusions

Social behavior depends on coordinated communication across hippocampal networks that encode social identity, including ventral hippocampus and CA2 and CA1 subfields, the ventral hippocampus–medial prefrontal cortex pathway that carries socially relevant information to prefrontal decision circuits, subcortical structures that encode internal state and hierarchy, and dopaminergic systems that assign motivational value to interaction. Across these nodes, stable neural networks, appropriate neuromodulatory inputs, and trophic support from BDNF–TrkB signaling are all critical for proper social memory and social motivation.

RTT, caused by pathogenic variants in *MECP2*, provides a case study of how this integrated system can fail. MeCP2 regulates activity-dependent transcription of *Bdnf* and many other genes involved in synaptic plasticity (44–46, 219). *Mecp2*-deficient mice show reduced BDNF levels in multiple brain regions, altered dendritic structure, impaired synaptic function, and atypical network dynamics (182, 185–187, 220). Behaviorally, these changes manifest as impaired social memory, atypical reciprocity, anxiety-like features, and altered dominance or aggression, often with preserved baseline sociability in simple tasks. This pattern reflects altered integration of social information with motivational value, which impairs flexible behavioral output in RTT individuals rather than a uniform reduction in social interest.

Several interventions in *Mecp2*-based RTT models, including enhancement of BDNF–TrkB signaling, restoration of MeCP2 expression after symptom onset, and stabilization of neuronal activity in defined projections, partially rescue physiological function, breathing, motor learning, and selected social behaviors (24, 42, 49, 145, 185–187, 220). These findings indicate that core RTT phenotypes reflect circuit-level dysregulation rather than irreversible developmental loss and highlight leverage points such as ventral hippocampus to prefrontal communication, prefrontal dopamine modulation during social reward seeking, and BDNF-dependent synaptic plasticity.

Taken together, this framework emphasizes that social behavior is both cognitive and motivational. It requires accurate recognition of others and the recall of past interactions, as well as appropriate assignment of value to those interactions and the capacity to act on that value. Embedding RTT within this framework links memory circuits to motivation circuits and provides a roadmap for future experiments. The objective shifts from describing global social deficits to developing targeted circuit-based strategies aimed at restoring social memory, social motivation, and ultimately meaningful social participation in neurodevelopmental disorders.
